# Development of a Real-Time Quantitative RT-PCR Assay for Detection of Bovine Rhinitis B Virus

**DOI:** 10.3389/fvets.2021.680707

**Published:** 2021-06-04

**Authors:** Yi-Lun Xie, Dian-Hong Lv, Xiao-Hui Wen, Qi Zhai, Man-Lin Luo, Wen-Kang Wei, Qin-Ling Chen, Shao-Lun Zhai

**Affiliations:** ^1^College of Veterinary Medicine, South China Agricultural University, Guangzhou, China; ^2^Scientific Observation and Experiment Station of Veterinary Drugs and Diagnostic Techniques of Guangdong Province, Ministry of Agriculture of Rural Affairs, Key Laboratory of Animal Disease Prevention of Guangdong Province, Institute of Animal Health, Guangdong Academy of Agricultural Sciences, Guangzhou, China; ^3^Agro-biological Gene Research Center, Guangdong Academy of Agricultural Sciences, Guangzhou, China

**Keywords:** bovine rhinitis B virus, RT-qPCR, TaqMan probe, detection, assay

## Abstract

Bovine rhinitis B virus (BRBV) has been frequently identified in cattle diagnosed with bovine respiratory disease complex (BRDC) in recent years, suggesting its potential contribution to BRDC. The goal of this study was to develop a TaqMan-based real-time quantitative RT-PCR assay for efficient BRBV detection. A pair of primers and a probe were designed based on the 3D gene of the BRBV genome. The assay was specific for BRBV and able to exclude bovine rhinitis A virus, foot-and-mouth disease virus and Senecavirus A. The limit of detection of the assay was 4.46 copies per reaction. A standard curve was plotted, with a coefficient of determination of 0.999 in the concentration range of 10^0^-10^8^ copies/μl. The reproducibility of the assay was acceptable, with the standard deviations of cycle threshold values lower than 1.00 in both intra- and inter-assay. Of 200 samples collected from 150 head of cattle in recent years in China, 11% (22/200) of the samples tested positive in the assay, i.e., 4.6% (7/150) of the cattle were BRBV positive. This study provides an efficient diagnostic tool for the epidemiological investigations of BRBV.

## Introduction

Bovine rhinitis B virus (BRBV), previously designated as bovine rhinovirus 2, is a non-enveloped single-stranded positive-sense RNA virus. According to the International Committee on Taxonomy of Viruses (1), BRBV, with bovine rhinitis A virus (BRAV), equine rhinitis A virus, and foot-and-mouth disease virus, are members of the *Aphthovirus* genus in the *Picornaviridae* family. BRBV was first isolated in 1971 in England and inferred to be a kind of BRAV (previously classified as bovine rhinovirus). Subsequently, it was differentiated from BRAV because of the absence of cross-reactions in the neutralization test against BRAV (2). It was found that BRBV infection could lead to slight pneumonia in specific pathogen-free calves (3). However, in the last decades, few reports onBRBV have been available. Bovine respiratory disease complex (BRDC) has been a widespread and damaging problem for the cattle industry (4). Recently, BRBV has been increasingly identified in metagenomics studies on cattle diagnosed with BRDC. In 2015–2019, such cases were reported in several countries including Canada (5), Mexico (6), Norway (7), Sweden (8), and the United States (9). Although the exact causative agent of BRDC remains uncertain, it is believed that numerous combinations of stressors could cause viral and bacterial infections that develop into BRDC (10). BRBV is believed to be one of the contributors, but exactly how BRBV participates in BRDC needs to be further investigated. In 2014, during another study in our laboratory, through next-generation sequencing, high reads of the BRBV nucleotide were unexpectedly mapped in a tissue homogenate of the lungs and spleen from a dead Holstein calf originating from Guangdong. Until then, BRBV had never been reported in China. Moreover, no detection assay specifically for BRBV has been reported so far. Conventional polymerase chain reaction (PCR) and reverse transcriptase polymerase chain reaction (RT-PCR) have been widely applied in field sample screening, but the forward and reverse primers provide limited specificity against sequences with close homology. TaqMan probe-based RT-qPCR, with an additional dual-labeled probe, has better specificity compared with conventional RT-PCR (11). To better understand the epidemiology of BRBV, and to provide a useful detection tool for further pathogenesis research, this study aimed to establish a rapid and reliable TaqMan probe-based real-time RT-qPCR assay. A pair of primers and a probe targeting the 3D gene in BRBV were designed, and the parameters were optimized. Evaluation tests were conducted, including sensitivity test, specificity test, reproducibility tests, plotting of the standard curve, and tests on clinical samples.

## Materials and Equipment

DNA/RNA extraction was conducted with the AxyPrep Plasmid Miniprep Kit (Union City, CA, USA) and AxyPrep Body Fluid Viral DNA/RNA Miniprep Kit (Union City, CA, USA). The RT-PCR kits used were as follows: TaKaRa PrimeScript™ One Step RT-PCR Kit Ver.2 (Otsu, Shiga, Japan) and TaKaRa One Step PrimeScript™ RT-PCR Kit (Otsu, Shiga, Japan). The gene cloning experiments were conducted with TaKaRa pMD19-T Vector Cloning Kit (Otsu, Shiga, Japan) and *E. coli* DH5α Competent Cells (Otsu, Shiga, Japan). The software used for sequence analysis were DNAStar 7.1 and MEGA 7.0. The software used for primes and probes design was Sigma-Aldrich OligoArchitect (St. Louis, MO, USA). The software used for RT-qPCR monitoring and results analysis was Analytik Jena qPCRsoft 4.0 (Jena, Germany). The apparatus used for RT-PCR was BIO-RAD T100™ Thermal Cycler (USA). The apparatus used for determination of DNA concentrations was Thermo Scientific NanoDrop Lite (Wilmington, DE, USA). The apparatus used for RT-qPCR was Analytik Jena qTOWER3/G (Jena, Germany). The DNA synthesis and sequencing were conducted by Sangon Biotech (Shanghai, China).

## Methods

### Primers and Probe

Nine BRBV complete coding sequences (CDSs) (GenBank Accession Numbers: EU236594, KP236130, KP264975, KP264980, KU159360, KU159361, KU168861, KU168862, and KY432299) obtained from GenBank were analyzed. A pair of primers and a probe were subsequently designed targeting the conserved region of the 3D gene with the online design tool and were synthesized. The primers (BRBV-144-F/R) and the probe (BRBV-144-Probe) are shown in Table 1. The size of the amplicon is 144 base pairs.

**Table 1:** Sequences and corresponding positions.

**Primers and probe**	**Sequence (5′-3′) and labels**	**Length (nucleotides)**	**Site relative to MT160419**
BRBV-144-F	CGTGGCACACTTCAGGAG	18	7,245–7,262
BRBV-144-R	GTGTACCCAYCTCARACGAAG	21	7,388–7,368
BRBV-144-probe	FAM-TRGCRGGTCTCGCTTTYCACAGT-BHQ1	23	7,276–7,298

*BRBV, bovine rhinitis B virus*.

### Preparation of Positive Control Plasmid

The BRBV-CHN1 (Accession Number: MT160419) isolate deposited in the laboratory was selected as the template for the positive control plasmid construction. A fragment of the genome was amplified through RT-PCR with primers BRBV-144-F and BRBV-144-R. The RT-PCR product was subsequently cloned into the pMD19-T vector (2,692 bp). The *E. coli* DH5α competent cells were transformed with the ligation product. The recombined pMD19-BRBV were extracted and sequenced. The plasmid DNA concentration was measured. The equation that was used to calculate the copy number of the pMD19-BRBV was as follows:

(1) $$\begin{array}{r} Number\;of\;copies\;per\;\mu l=\frac{c\times 6.022\times 10^{23}}{n\times 660} \end{array}$$

where *c* is the concentration of pMD19-BRBV (g/μl), *6.022* × *10*^23^ is the Avogadro constant, *n* is the number of base pairs in a single pMD19-BRBV, and *660* (Da) is the average weight of a dsDNA base pair.

### Optimization of Reverse Transcriptase Quantitative Polymerase Chain Reaction for Bovine Rhinitis B Virus

The reaction mixture was prepared, and conditions were set according to the kit instructions. The initial 25-μl reaction mixture contained 1 μl of PrimeScript One Step Enzyme Mix, 12.5 μl of 2× One Step Buffer, 1.0 μl of BRBV-144-F, 1.0 μl of BRBV-144-R, 1.0 μl of BRBV-144-Probe, 2.0 μl of template DNA/RNA, and 6.5 μl of RNase-free dH_2_O. The initial reaction program was as follows: reverse transcription at 50°C for 30 min, predenaturation at 94°C for 2 min, 45 cycles of 98°C for 10 s, 58°C for 30 s, and scanning for the fluorescence signal at the final stage in each cycle.

The concentration of the forward and reverse primers was first to be optimized among 0.2, 0.4, 0.6, 0.8, and 1.0 μM at an initial annealing temperature of 58°C, initial concentration of the probe of 0.2 μM, and 4.46 × 10^2^ copies of pMD19-BRBV. Then, the concentration of the probe was optimized among 0.2, 0.4, 0.6, 0.8, and 1.0 μM, at an annealing temperature of 58.0°C, optimized concentration of the primers, and 4.46 × 10^4^ copies of pMD19-BRBV. Next, the annealing temperature was optimized using the optimized concentration of the primers and the probe and 4.46 × 10^2^ copies of pMD19-BRBV. The optimization ranges of annealing/extension temperatures were 57.0, 58.0, 59.0, 60.0, 61.0, 61.9, 62.5, and 62.7°C. All optimization tests were conducted in three replicates. The threshold was set by default. The optimal reaction parameters were determined by the cycle threshold (C_T_) value and the fluorescence intensity. The sensitivity tests, quantification experiment, specificity tests, reproducibility tests, and sample tests in this study were conducted at the optimized reaction conditions.

### Sensitivity Test and Standard Curve

The pMD19-BRBV was serially diluted 10-fold for 12 times. The sensitivity test was performed in three replicates on a range of dilutions from 2.23 × 10^−3^ to 2.23 × 10^8^ copies/μl. The limit of detection was defined as the lowest pMD19-BRBV copy number for which at least one replicate tested positive. Every outcome was determined to be positive where a valid C_T_ value occurred. C_T_ values were valid when only the corresponding plotting of the absolute fluorescence values were sigmoidal curves and agreed with the kinetics of amplification. The pMD19-BRBV dilutions of 2.23 × 10^0^ to 2.23 × 10^8^ copies/μl were run by the optimized RT-qPCR assay and replicated eight times to establish a standard curve, which was calculated and plotted automatically by qPCRsoft 4.0.

### Specificity Test

The assay was tested with the nucleic acid extracts of Senecavirus A, foot-and-mouth disease virus, border disease virus, bovine coronavirus, bovine viral diarrhea virus, and influenza D virus. Border disease virus and bovine viral diarrhea virus were kindly provided by Dr. Mao from the Institute of Veterinary Medicine, Jiangsu Academy of Agricultural Sciences, Nanjing, China. Senecavirus A, bovine coronavirus, and influenza D virus were isolated and preserved in our laboratory. The nucleic acid of the foot-and-mouth disease virus was obtained from a commercial vaccine. The specificity test was conducted on the extracts. BRAV was specifically added to the specificity test because of its homology with BRBV. Similarity analysis showed that the sequences of the primers and the probe in this study resembled that of the corresponding region of BRAV (maximum identity with BRBV-144-F: 61.1%, with BRBV-144-R: 81.0%, and with BRBV-144-Probe: 65.2%). The corresponding region of one BRAV isolate (Accession Number: KP236128; sites: 7,230–7,442) that shared the highest similarity with that of the primers and the probe in this study was synthesized, and the product pUC57-BRAV was tested with the optimized RT-qPCR developed in this study. The specificity test on BRAV was replicated four times on the same occasion and repeated two times on different occasions. Positive and negative controls were included in each run.

### Reproducibility Test

The reproducibility test consisted of intra- and inter-assay. The DNA templates that were used in the reproducibility test were pMD19-BRBV dilutions of 2.23 × 10^2^, 2.23 × 10^4^, and 2.23 × 10^6^ copies/μl. Intra-assay was carried out by running the assay on the three dilutions with five replicates for each dilution in one run. Inter-assay was carried out by running the assay on the three dilutions at a time and repeating another four times on four different occasions.

### Test With Clinical Samples

A total of 200 samples (including tissue homogenate, nasal swabs, blood, content in digestive tract, and feces) were collected from cattle in Guangdong, Hunan, Hainan provinces from 2014, 2019, and 2020. Two of the samples were collected from a dead Holstein calf in Guangdong in 2014, one of which was a homogenate of the lungs and spleen, and the other was a fecal sample. These samples were extracted and tested with the optimized RT-qPCR assay. More than one sample was collected from some cattle. The total number of cattle involved was 150. Positive and negative controls were included in each run. Eight of the valid positive samples were randomly chosen to be amplified through RT-PCR with BRBV-144-F and BRBV-144-R and sequenced to examine any false-positive reaction.

## Results

### Optimized Conditions for Reverse Transcriptase Quantitative Polymerase Chain Reaction for Bovine Rhinitis B Virus

The optimal concentration of the primers BRBV-144-F and BRBV-144-R was 0.8 μM (Table 2). The optimal concentration of the probe BRBV-144-Probe was 0.6 μM (Table 2). The optimal annealing temperature and its duration was 58°C for 30 s (Table 3).

**Table 2:** Optimization of primes and probe.

**Concentration (μM)**	**Mean C_**T**_ value of primer optimization (*n* = 3) ± standard deviation**	**Mean C_**T**_ value of probe optimization (*n* = 3) ± standard deviation**
0.2	29.49 ± 0.77	22.66 ± 0.30
0.4	28.60 ± 0.18	21.78 ± 0.35
0.6	28.43 ± 0.06	21.56 ± 0.32
0.8	28.42 ± 0.20	22.57 ± 0.09
1.0	29.10 ± 0.16	23.65 ± 0.55

**Table 3:** Optimization of annealing temperature.

Annealing temperature (°C)	57.0	58.0	59.0	60.0	61.0	61.9	62.5	62.7
Mean C_T_ value (*n* = 3) ± standard deviation	25.56 ± 0.27	26.02 ± 0.08	26.15 ± 0.06	26.14 ± 0.24	26.35 ± 0.39	27.06 ± 0.28	27.36 ± 1.05	27.19 ± 0.77

### Sensitivity and Specificity

The DNA concentration of the undiluted pMD19-BRBV was 69.37 ng/μl, i.e., 2.23 × 10^9^ copies/μl. The lowest copy number that gave any valid positive result was 2.23 × 10^0^ copies/μl (all three replicates had valid C_T_-values; maximum C_T_ = 38.23), i.e., the limit of detection was 4.48 copies per reaction. As for the specificity test, none of the selected viruses other than BRBV tested positive.

### Standard Curve and Reproducibility

**Figure 1:**
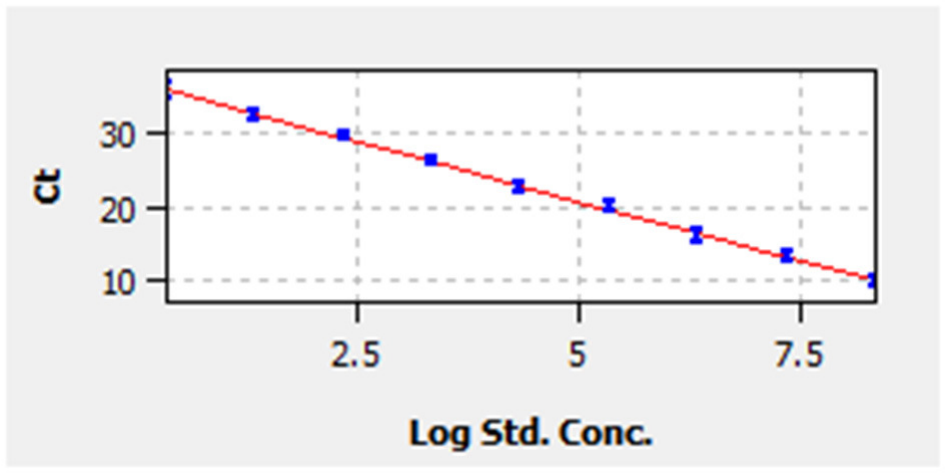
Standard curve. Correlation between cycle threshold value (y-axis) and the logarithm of concentration of 10-fold-diluted pMD19-bovine rhinitis B virus (BRBV) (x-axis). The x-axis in the graph starts from the logarithm of concentration of 2.23 × 10^0^ copies/μl. The standard equation is y = −3.29 × Lg Con. + 37.17.

**Figure 2:**
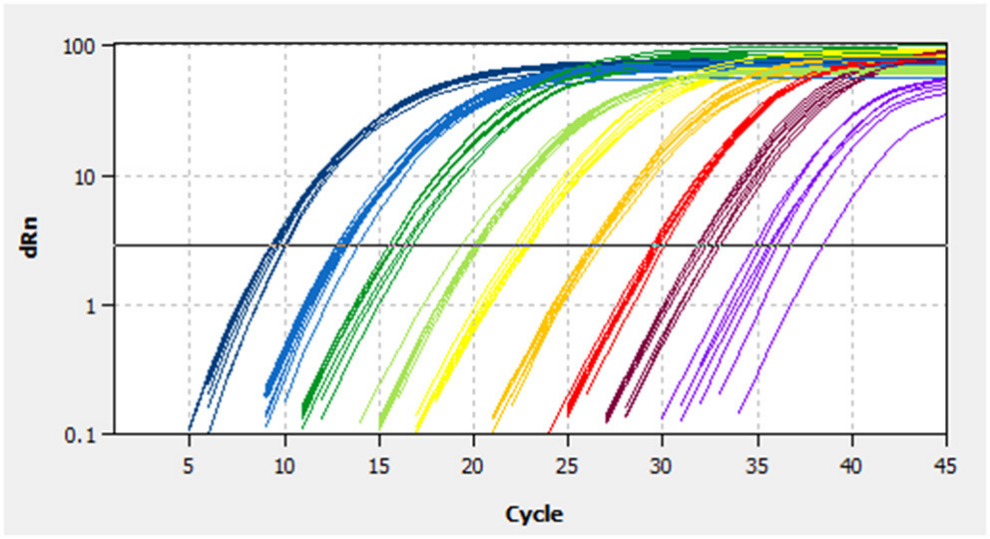
Fluorescence spectra for the standard curve. Fluorescence curves of different colors represent different concentrations of pMD19-BRBV. The concentrations were 2.23 × 10^8^ to 2.23 × 10^0^ copies/μl from left to right, respectively. dRn, the fluorescence intensity data standardized to the value 100 for highest fluorescence intensity.

A linear standard curve (Figure 1) with a slope of −3.29 was generated. With the starting concentration set to 2.23 × 10^8^ (copies/μl), the intercept of the standard curve was 37.17. The coefficient of determination (*R*^2^) was 0.999, and the PCR efficiency was 101%. The logarithmic graph of relative fluorescence value for the standard curve extrapolation is shown in Figure 2. The reproducibility test results are as shown in Table 4, with all SDs of C_T_ values lower than 1.00 in both intra- and inter-assay.

**Table 4:** Reproducibility of reverse transcriptase quantitative polymerase chain reaction (RT-qPCR).

	**Mean C**_**T**_ **values ± standard deviation (*****n*** **= 5)**	
**Concentration of pMD19-BRBV (copies/μl)**	**Intra-assay**	**Inter-assay**
2.23 ×10^2^	31.52 ± 0.36	31.17 ± 0.16
2.23 ×10^4^	24.36 ± 0.43	24.54 ± 0.97
2.23 ×10^6^	15.50 ± 0.55	14.51 ± 0.38

### Clinical Samples

**Figure 3:**
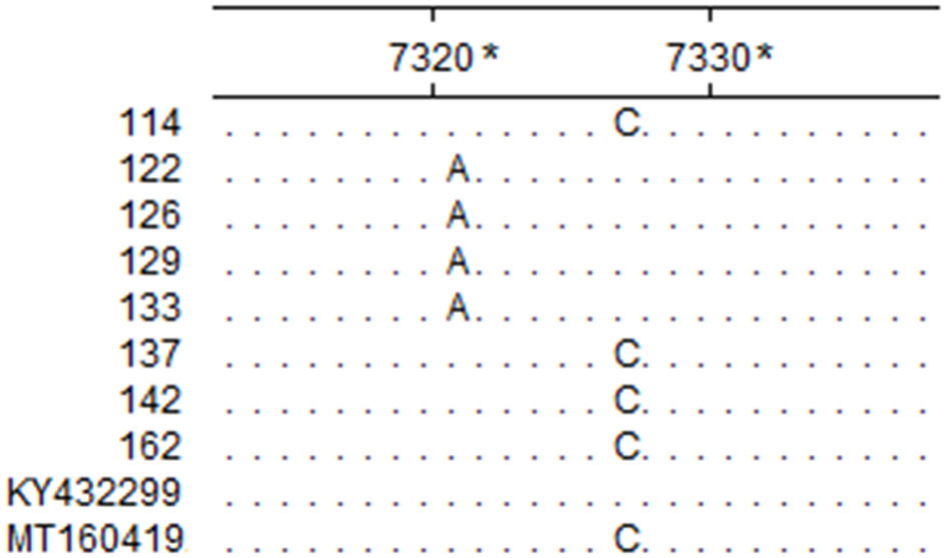
Partial sequence alignment of the eight positive samples and reference isolates. The nucleotides in each site that are identical in the eight positive samples and reference isolates BRBV-SWE1 (Accession Number: KY432299) and BRBV-CHN1 (Accession Number: MT160419) are represented with a dot. *The sites are relative to BRBV-SWE1 (Accession Number: KY432299).

The assay developed in this study was tested with clinical samples, and the results showed that 11% (22/200) of the samples tested positive, which were one homogenate of the lungs and spleen, three stool samples, and 18 nasal swabs. Among the cattle, 4.6% (7/150) tested positive (Table 5). The sequencing results of the eight positive samples showed that all eight sequences of RT-PCR products matched that of BRBV, among which four sequences possessed a mutation site that differed from the pMD19-BRBV template (Figure 3).

**Table 5:** Information about positive samples.

**Year**	**Location of collecting (province)**	**Origin of cattle (province)**	**Type of positive sample**	**Quantity of positive samples/total number of all types of samples**	**Quantity of positive cattle/total number of cattle**
2014	Guangdong	Guangdong	Homogenate of lungs and spleen	1/2	1/1
2019	Hunan	Guizhou	Nasal swabs	2/70	2/69
2020	Hunan	Shanxi	Nasal swabs and feces	19/128	4/80

## Discussion

**Figure 4:**
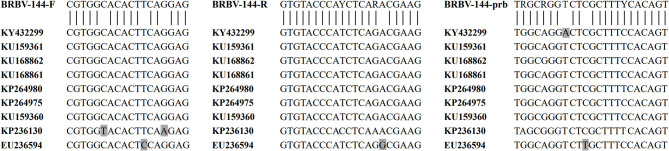
Alignment of the primers and the probe with BRBV isolates. The vertical bars below the sequences of primers/probe and above the sequences of the BRBV isolates represent the complete compatibility for the primers and the probe with the selected BRBV isolates. The nine sequences of BRBV isolates in the figure were retrieved from GenBank. Nucleotides that differ from the consensus are marked with a gray background.

The genetic divergence among BRBV isolates is considerable. Currently, there are 16 different partial or complete CDSs published in the GenBank database, yet only nine of them were complete. Sequence analysis showed that the nine complete CDSs shared poor identity pairwise (77.1–98.5%; mean identity: 83.21%), which indicated that the design of primers and probes would be challenging. To meet this challenge, the nine complete CDSs were aligned, and it was found that the alignment of the 3D gene showed better consistency with fewer mutations, so it was chosen for the initial design templates. Furthermore, degenerate bases (R = A/G; Y = C/T) were employed for the primers, and the probe in this study to increase the flexibility against sequences from different BRBV isolates was deposited in GenBank (Figure 4). Although field sample tests were conducted, the samples lacked diversity in space and time, and thus, epidemiological presumptions cannot be made. However, it is noteworthy that three fecal samples from Hunan in 2020 tested positive, while the fecal sample from Guangdong in 2014 tested negative. The C_T_ values of the positive fecal samples were no higher than that of the nasal swabs. Since the positive fecal samples were collected on the ground around the cattle, while the fecal sample in 2014 was collected straight from the anus of the cattle, it is deduced that the feces on the ground were contaminated by nasal secretions from the cattle. However, it is still possible that the cattle swallowed the respiratory tract secretions and the viruses passed the intestines. There was evidence that supported the applicability of the assay developed in this study. As shown in the sequence alignment result in Figure 3, samples 122, 126, 129, and 133 simultaneously possessed a mutation that matched neither that of BRBV-CHN1 (Accession Number: MT160419) and BRBV-SWE1 (Accession Number: KY432299). This means that the positive cattle were likely infected by an isolate that differed from BRBV-CHN1 and BRBV-SWE1, and the isolate was identified with the assay developed in this study. Nevertheless, given the variability of the viruses from *Picornaviridae* and the lack of abundance of the present BRBV nucleotide sequences on record, the assay might not be as applicable as new isolates increase. However, it is still a faster and more affordable approach to detecting BRBV compared with next-generation sequencing, especially in epidemiological studies. In addition, the assay developed in this study could also be applied for quantification in pathogenesis research. In the current study, an RT-qPCR assay targeting the 3D gene in BRBV was developed and evaluated. The evaluation results showed that the assay had great sensitivity with a limit of detection of 4.46 copies per reaction. No false-positive reaction occurred with the selected common bovine viruses and viruses from *Picornaviridae*. The reproducibility was acceptable with SDs of C_T_ values lower than 1.00 in the intra- and inter-assay. These results suggested that the developed RT-qPCR is practical and dependable for BRBV detection, and it is expected to be of benefit in epidemiological and pathogenesis studies of BRBV.

## Data Availability Statement

The datasets presented in this study can be found in online repositories. The names of the repository/repositories and accession number(s) can be found in the article/supplementary material.

## Author Contributions

Y-LX, D-HL, X-HW, and S-LZ carried out the conceptual and experimental work. Y-LX and S-LZ wrote the first draft of the manuscript. QZ and W-KW contributed to the writing and review of the manuscript. Q-LC, S-LZ, and M-LL supervised the study. All authors have approved the manuscript for publication.

## Conflict of Interest

The authors declare that the research was conducted in the absence of any commercial or financial relationships that could be construed as a potential conflict of interest.

## References

[1] International Committee on Taxonomy of Viruses. Taxonomy. (2019). Available online at: https://talk.ictvonline.org/taxonomy/ (accessed October 16, 2020).

[2] Reed SE Tyrrell DAJ. Studies on a rhinovirus (EC11) derived from a calf I. Isolation in calf tracheal organ cultures and characterization of the virus. J Comp Pathol. (1971) 81:33–40. 10.1016/0021-9975(71)90052-1 4326295

[3] Betts AO Edington N Jennings AR Reed SE. Studies on a rhinovirus (EC11) devived from a calf. II. Disease in calves. J Comp Pathol. (1971) 81:41–8. 10.1016/0021-9975(71)90053-3 4326296

[4] Hause BM Collin EA Anderson J Hesse RA Anderson G. Bovine rhinitis viruses are common in U.S. cattle with bovine respiratory disease. PLoS ONE. (2015) 10:1–12. 10.1371/journal.pone.0121998 25789939 PMC4366061

[5] Zhang M Hill JE Fernando C Alexander TW Timsit E van der Meer F. Respiratory viruses identified in western Canadian beef cattle by metagenomic sequencing and their association with bovine respiratory disease. Transbound Emerg Dis. (2019) 66:1379–86. 10.1111/tbed.13172 30873724 PMC7168561

[6] Mitra N Cernicchiaro N Torres S Li F Hause BM. Metagenomic characterization of the virome associated with bovine respiratory disease in feedlot cattle identified novel viruses and suggests an etiologic role for influenza D virus. J Gen Virol. (2016) 97:1771–84. 10.1099/jgv.0.000492 27154756 PMC5772826

[7] Myrmel M Oma V Khatri M Hansen HH Stokstad M Berg M. Single primer isothermal amplification (SPIA) combined with next generation sequencing provides complete bovine coronavirus genome coverage and higher sequence depth compared to sequence-independent single primer amplification (SISPA). PLoS ONE. (2017) 12:e0187780. 10.1371/journal.pone.0187780 29112950 PMC5675387

[8] Blomström AL Oma V Khatri M Hansen HH Stokstad M Berg M. Genome sequence of a bovine rhinitis B virus identified in cattle in Sweden. Genome Announc. (2017) 5:5–6. 10.1128/genomeA.00172-17 28495761 PMC5427196

[9] Ng TFF Kondov NO Deng X Van Eenennaam A Neibergs HL Delwart E. A metagenomics and case-control study to identify viruses associated with bovine respiratory disease. J Virol. (2015) 89:5340–9. 10.1128/jvi.00064-15 25740998 PMC4442534

[10] Peel DS. The effect of market forces on bovine respiratory disease. Vet Clin North Am Food Anim Pract. (2020) 36:497–508. 10.1016/j.cvfa.2020.03.008 32451038

[11] Heid CA Stevens J Livak KJ Williams PM. Real time quantitative PCR. Genome Res. (1996) 10:986–94. 10.1101/gr.6.10.986 8908518

